# Food Adulteration

**Published:** 1887-09

**Authors:** 


### SELECTIONS.

### Food Adulteration.

A recent issue of the New York *World* contained a list of adulterants found in articles of food and drink, which is instructive reading to the public generally, and to sanitarians particularly. It is of interest to every dweller in a civilized community, and of quite as much importance to this country as its commerce, the improvement of its rivers and harbors, or any question of coast defense. On reading the list one is amazed at the ingenuity and dishonesty of civilized Christian man. The majority of our foods and condiments, of our articles of drink, are so adulterated that it may now well be said: he is a wise man who knows what he is eating. It has not been very long since we commented on this subject, but so long as the evil exists it is of importance that repeated attention be called to it. Below we give the list as printed in the *World:*

Coffee (ground).—Chicory, peas, beans, date-stones, biscuits, figs, roasted hominy, burnt sugar, acorns, mangel-wurzel, dandelion, turnips, parsnips, carrots, and rye and potato flours.

Tea.—Exhausted tea leaves (faced or colored and fixed up with plumbago, gum, indigo, Prussian blue, turmeric, China clay, mica, soapstone or French chalk, sulphate of lime, rose pink, Venetian red, carbonate of copper, arsenite of copper, chromate and bi-chromate of potash, and carbonate of lime and magnesia), leaves of the elder, willow, sloe, and other plants and trees, lie tea, paddy husk, sweepings of the tea-house floors, sand, quartz, starch, and magnetic oxide of iron.

Sugar (cane).—Grape or starch glucose, wheat and potato flours, tapioca starch, blood, pipeclay, marble dust, gypsum, bone dust, lead, iron, lime, sand.

Cocoa and chocolate.—Arrow-root, sago, starch, chicory, ferruginous earths, chalk, oxide of iron, sulphate of lime, wheat and Indian flours, tapioca, cocoa husks, old sea biscuit, potatoes, molasses, and animal fats, such as tallow and lard.

Wheat flour.—Adulterated with inferior or old flour, potato flour, ground beans, peas, rye, barley and rice, alum, chalk, gypsum, soapstone, and carbonate of magnesia.

Buckwheat.—Wheat and other flours.

Honey.—Cane sugar, chalk, sulphate of lime, pipe clay, gypsum, and glucose.

Lard.—Oleomargarine, cotton-seed oil, potato flour, water, mutton suet, alum (for color), carbonate of soda (for taste), caustic soda (for taste or smell).

Cream tartar.—Gypsum, starch, acid-phosphate of calcium, tartaric acid.

Saleratus.—Most of what is now sold as saleratus, is bi-carbonate of soda.

Bi-carbonate of soda, or baking soda.—Gypsum, sulphate of soda, carbonate of lime.

Sugar confectionery.—Glucose, terra alba, chalk, arrowroot, sand, wheat and pototo flour, hydrated sulphate of lime, with (for coloring) cochineal, lake, indigo, and Prussian blue, carbonate of copper, carbonate of lead or white lead, vermilion, gamboge, chromates of lead, sap green, arsenite of copper, Indian red, umber, sienna, Vandyke brown, cobalt, smalt, litmus, Naples yellow, bi-sulphuret of mercury, sulphuret of arsenic, bronze powders or alloys of copper and zinc.

Mustard.—Flour, turmeric, cayenne pepper, ginger, plaster of paris, linseed meal, radish seeds, chromate of lead, gypsum, sand.

Oatmeal.—Barley meal, rice and corn flour, ground husks of the cereals.

Wheat bread.—Barley, oat, and pea flours, bone dust, carbonates of lime, magnesia and soda, sulphate of copper and lead chromates (for color), alum (for taste).

Cheese.—Oleomargarine, lard, cotton-seed oil, borax, saltpetre, potatoes, beans, soapstone, soda, potash, urine, sulphate of zinc, blue vitriol, arsenic (in foreign cheese).

Butter.—Oleomargarine, cheaper fats, cotton-seed oil, alum, borax, barium, chalk, flour, gypsum, lead carbonate, yellow lead chromate, potato flour, salt, soapstone, starch, and sodium silicate or soluble glass.

Arrow-root.—Sago, potato and tapioca starches, and ground rice.

Cinnamon.—Cassia and most of the other spices, flour, meal, and arrow-root.

Pepper.—Ground rice and beans, mustard husk, salt, oil-cake, and clay, and for colors red lead, vermilion or bi-sulphuret of mercury, Venetian red, turmeric, and charcoal

Ginger.—Wheat, sago, and potato flour, ground rice, mustard husks, turmeric.

Maple sugar.—Cane sugar, glucose, and flour.

Chicory.—Roasted wheat and rye flour, burnt beans and acorns, carrots, mangel-wurzel, roasted biscuit, sawdust, and oak-bark tan.

Sago.—Potato flour.

Vinegar.—Water, with burned sugar for color, sulphuric acid, acetic, hydrochloric, nitric, and tartaric acids, cayenne pepper, salt, and mustard seed.

Pickles.—For coloring, acetate of copper.

Sauces.—Chalk and plaster of Paris, and for coloring red earths.

Marmalade.—Pulp of apple or turnip.

Olive oil.—Cotton-seed oil and the cheaper oils, of the poppy, peanut, grape seed and beachnut, and coal oil, with lead to correct rancidity and copper for a greenish color.

Brandy.—Water, corn spirits, molasses, burnt sugar (for color). Artificial brandy made from other spirits and flavoring extracts is common.

Gin.—Water, cayenne, flavoring extracts, slum, and salt of tartar (for fining), sulphuric acid, coriander seed, oil of almonds, calamus root, orris root, orange peel, acetate of lead, oil of turpentine, and gray and white salts for taste or smell.

Rum.—Water, cayenne, burnt sugar, and cocculus indicus for taste or smell.

Whiskey.—Principally water, cayenne pepper, bitter almonds, tonka bean, burnt dried peaches, beet root, and a variety of flavoring extracts, sulphuric acid, creasote, and sweet spirits of nitre.

Wines.—Mixtures of inferior wines, cider, and the juices of rhubarb, gooseberries, and pears, with (for coloring) logwood, elderberry juice, Brazilwood, billberries, burnt sugar, black cherries, and cochineal, and for taste and smell, sulphate of potash, bi-tartrate of potash, lead, oak, sawdust, catechu, cherry-laurel water, carbonate of soda, and artificial flavorings.

Ale, beer, and porter.—Water, with, for taste and smell, cocculus indicus, opium, cayenne, ginger, quassia, colocynth, caraway and coriander seeds, orange powder, honey, licorice, sulphate of iron, sulphuric acid, cream of tartar, camomile, alum, carbonate of potash, nitric acid, horehound, blessed thistle, sweet flag, gentian, aloes, molasses, juniper berries, burnt flour, isinglass, albumen, ivory, black gypsum, salt, glycerine, salicylic acid, decoction of calves' feet, glue, copperas, henbane, belladonna leaves, nux vomica, hartshorn, strychnine, tobacco, shavings and oyster shells.

Milk.—Water principally, flour or starch, boiled white carrots, milk of almonds, sheep's brains, gum tragacanth, carbonate of soda, chrome yellow for colloring.

As medical men we are concerned with the detection and punishment of dishonesty only when its results are injurious to health. It requires no great amount of argument, however, to show that food adulteration is prejudicial to health. From causes which we need not stop to mention the American public has come to be too lenient with the dishonesty which shields itself under the name of " business." Such laws as we have for the protection of health and life are, as a rule, improperly enforced. As self-constituted conservators of public health should we not bring this matter before our legislative bodies—not once, but repeatedly—until this disgrace is wiped out from the country?

---

Can there be any wonder after reading the above list that we are a nation of dyspeptics? While it may be said that many of the adulterants of our foods are innocuous, it must be admitted that others, taken repeatedly, even if in small quantities, are injurious, and are certainly not foods in the common acceptation of the term. For example, an enormous quantity of tea is consumed in this country, and a reference to the list will show that tea is more largely and more perniciously adulterated than any other article in such common use, except perhaps malt liquors. The consumption of sugar confectionery by children is very large, and the adulterants of this article are by no means harmless. And when it is remembered that milk is the principal food of a very large proportion of the population (young children) who can take no other food, the possible and almost certain injury may well cause us to endeavor, by every possible means, to throttle an evil which is worse than the plague.—*Journal American Medical Association.*

---

THE necessity of care to protect teeth from injury when administering tincture of iron is shown by the immersion of a tooth in a 11-per-cent. solution of tincture of iron. The enamel of such a tooth is entirely destroyed in one hour. Druggists should take pains to caution customers against this danger.

